# The Potential Effects of Diffuse Scleroderma in a Patient With Cervical Kyphosis

**DOI:** 10.7759/cureus.64236

**Published:** 2024-07-10

**Authors:** Haider Ghumman, Jeffrey Farooq, Puya Alikhani

**Affiliations:** 1 Neurosurgery and Brain Repair, University of South Florida Morsani College of Medicine, Tampa, USA

**Keywords:** rheumatology, autoimmune, scleroderma, cervical kyphosis, spinal fusion

## Abstract

Scleroderma is a complex autoimmune disorder that primarily affects the connective tissue. Its key pathogenesis comprises vascular abnormalities, autoimmunity, and tissue fibrosis. While the exact etiology of the disease is unclear, patients may exhibit a wide array of symptoms. Scleroderma can rarely induce systemic effects that alter normal cervical spine anatomy. The effects on the cervical spine may be mediated through autoimmune phenomena or dystrophic calcinosis along the vertebral column. We discuss a rare case involving a 60-year-old female with a four-month history of scleroderma, who presented with cervical kyphosis, neck pain, impaired ambulation, dysphagia, edema, and reduced range of motion.

## Introduction

Scleroderma is an autoimmune connective tissue disorder with a complex pathophysiology. Though there are various forms of scleroderma, it may be fundamentally classified into two primary forms: localized and systemic. Localized scleroderma affects the skin and subcutaneous tissue, while systemic scleroderma extends into the internal organs and is accompanied by Raynaud’s phenomenon [1,2]. Both forms are characterized by increased collagen synthesis through the differentiation of activated fibroblasts into myofibroblasts [1]. Current theories support the hypothesis that an initial vascular insult mediated by viral particles, environmental exposures, proteolytic enzymes, or inflammatory cytokines initiates a cascade, resulting in tissue fibrosis [3]. Thus, three mechanisms underlie the classical pathogenesis of scleroderma - vascular abnormalities, autoimmunity, and tissue fibrosis - and may contribute to the rare neurological sequelae observed in scleroderma patients [3].

Despite its well-characterized systemic effects, scleroderma rarely affects the cervical spine. Soft tissue calcification predominantly occurs at pressure points, such as the wrists, elbows, and knees, or in areas of trauma. However, dystrophic calcinosis may develop in the spinal column in scleroderma patients [4-6]. Cervical spinal calcinosis produces appreciable neurological symptoms via spinal canal stenosis and spinal instability, although the mechanism underlying this calcification is not well understood [4,7]. The onset of neurological symptoms may be mediated by compression or entrapment of spinal nerves exiting through the spinal foramen, leading to radiculopathy or myelopathy [8]. Scleroderma may also present with osteolysis of skeletal bone. Though this generally occurs in the distal phalanges, osteolysis has also been observed in other areas as well, such as the ribs, mandible, distal clavicle, and cervical spine [9,10]. While researchers have previously established the presence of cervical calcification and osteolysis in scleroderma patients [4,7,8,11-14], cervical kyphosis has rarely been reported [14]. We present a rare case of cervical kyphosis and cervical spine abnormalities in a scleroderma patient.

## Case presentation

**Figure 1:**
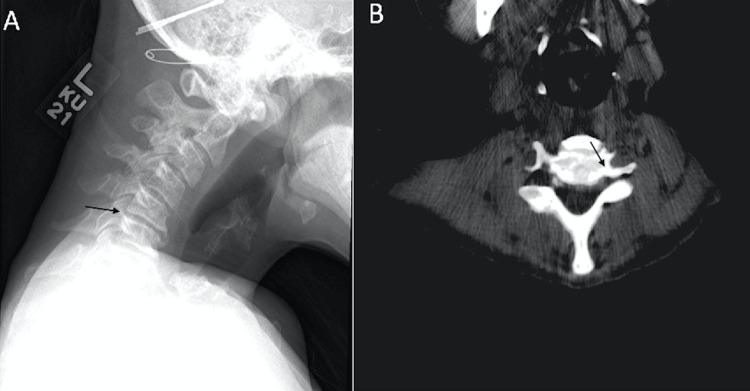
A) Lateral X-ray of the cervical spine. B) Transverse non-contrast cervical CT scan CT: computed tomography

A 60-year-old female patient with a four-month history of diffuse scleroderma presented with severe cervical kyphosis with chin-to-chest deformity, worsening neck pain, an inability to ambulate, and difficulty opening her mouth. Physical exam revealed bilateral facial, upper extremity, and lower extremity edema with impaired digital and hip flexion. Labs were significant for normocytic anemia and elevated C reactive protein. A cervical X-ray demonstrated degenerative loss of disc height at C5-C6 and C6-C7 (Figure 1A). A non-contrast cervical CT scan showed C3-C4 with a diffuse disc bulge and posterior osteophytes with mild canal narrowing (Figure 1B).

There were facet hypertrophic changes with mild bilateral neural foraminal narrowing. C4-C5 also exhibited mild degenerative changes, canal narrowing, uncovertebral osteophytes, and narrowing of the bilateral neural foramen. C5-C6 showed diffuse disc bulging with moderate canal stenosis, uncovertebral osteophytes, and facet hypertrophic changes. There was modest left neural foraminal narrowing and minimal right neural foraminal canal narrowing. C6-C7 also demonstrated a mild bulge asymmetric to the left with mild canal narrowing and mild left neural foraminal narrowing. The osteoarthritic and degenerative disc changes were most prominent at C5-C6.

The decision was made to proceed with a posterior approach kyphosis correction to circumvent significant secretions that would require frequent suctioning. The patient was placed under general anesthesia and positioned prone for a subfascial dissection from C2 to T8. Dissection was complicated by extensive connective tissue thickening and adequate exposure of the bony elements was protracted. After successful dissection, all bony elements were decorticated and C2 to T8 fusion was performed with bilateral screw placement at the level of C2-C6 and T1-T8. Allografts and autografts were placed bilaterally at each level. Ponte osteotomies were performed at T2-T3, T3-T4, T4-T5, and T5-T6. Neuromonitoring was utilized throughout the procedure to observe somatosensory-evoked potentials and motor-evoked potentials, which both remained at baseline for the duration of the surgery.

Postoperatively, the patient continued to display severe pharyngeal dysphagia requiring frequent oral care, including suctioning. She was incontinent of both bowel and stool throughout the hospital course and cycled through periodic episodes of confusion with grossly stable vital signs. However, on postoperative day 14, she became pyretic, tachypneic, tachycardic, and hypotensive with a dramatic decrease in mentation such that she could not respond to verbal commands. She was started on empiric sepsis treatment and a skin biopsy was taken to investigate concerns of paraneoplastic scleroderma. A percutaneous endoscopic gastrostomy tube was inserted on postoperative day 21 and the patient began to stabilize. On postoperative day 30, she tested positive for coronavirus disease 2019 (COVID-19), complicating her recovery. The underlying etiology explaining her multi-system presentation was ultimately diagnosed by dermatology and pathology as diffuse scleroderma.

## Discussion

Scleroderma is an autoimmune disorder characterized by antibodies targeting connective tissues and blood vessels [2]. The hallmark of scleroderma is skin thickening due to fibrosis of underlying connective tissue [15], which made it challenging in our patient to adequately expose the bone during the subfascial dissection from C2 to T8, prolonging the length of the procedure. However, the pathogenesis of scleroderma in the patient was unique due to changes in vertebral stability and the development of neurological sequelae. Though unclear, the findings in this patient may also be attributed to age-related changes and degeneration with diffuse scleroderma playing a role in vertebral stability.

We present a rare case of cervical kyphosis and cervical spine abnormalities in a scleroderma patient. Previous studies have examined the effects of scleroderma on the vertebral column [4,7,8,11-14], establishing a correlation between calcifications along the cervical spine and neurological symptoms. Calcification of the cervical spine may lead to the narrowing of the spinal canal and neural foramen, causing entrapment of nerves within the spinal canal or those exiting through the neural foramen. The entrapment of these nerves may contribute to the neurological presentations of scleroderma patients with dystrophic calcifications along the cervical spine.

Despite the wide range of scleroderma presentations, patients with cervical spine calcifications present with well-defined symptoms. Table 1 outlines the symptoms, imaging, and treatment of previously reported cases of scleroderma patients with dystrophic calcifications of the cervical spine. The most common sequelae include pain [7,12-14], dysphagia [7,11-14], weakness [4,7,8,12], and difficulty with ambulation [4,8,9,13]. Our patient presented with all of these symptoms, highlighting the possible role of cervical calcifications in her neurological deficits. Interestingly, these calcifications were not visible in the patient's imaging, yet the symptoms align with scleroderma patients with established calcifications. Though previous studies indicate the presence of calcified masses near cervical vertebrae, a large majority of patients did not have associated cervical kyphosis. Cervical kyphosis may be secondary to a wide range of underlying etiologies, yet its association with scleroderma is unclear. It is important to manage cervical kyphosis appropriately, as it may contribute to neurological deficits such as myelopathy, radiculopathy, pain, and dysphagia [16]. Though cervical kyphosis may be corrected surgically or non-surgically, the underlying etiology of cervical kyphosis in some patients may be linked to scleroderma, necessitating its management in addition to cervical spine correction.

**Table 1:** Reported cases of scleroderma with dystrophic calcifications of the cervical spine BUE: bilateral upper extremity; BLE: bilateral lower extremity; N/A: not applicable

Study	Age, years	Sex	Presentation	Imaging	Treatment
Nguyen et al. [4]	57	F	BUE/BLE weakness; gait imbalance; limited mobility	Calcified soft-tissue masses at C4-C5 and C2-C3; subluxation at C4-C5	Laminectomies at C4-C7; fixation and fusion at C3-T1
Faraj et al. [7]	66	F	Back pain radiating to leg; dysesthesia at S1; dysphagia; Raynaud’s phenomenon	Masses at C6-C7 and C7-T1; subluxation throughout the cervical spine; calcinosis at L5-S1	Cervical mass resection, decompression; laminectomy, PLIF, and posterolateral fusion at L5-S1
Karschnia et al. [8]	58	F	Upper abdomen pain; progressive hand weakness, dysesthesias; ambulatory dysfunction; dysphagia; Raynaud’s phenomenon	Soft tissue calcification posterior to C4	Laminectomy at C4-C6; mass removal fusion at C2-T1
Bluett et al. [11]	64	F	Dysphagia; Raynaud’s phenomenon	Calcinosis with spondylolisthesis at C3-C4	Long-term rheumatological care
Smucker et al. [12]	60	M	Neck pain; hand dysfunction; stumbling gait; dysphagia	Calcific masses at C1-C2; subluxation at C2-C3	Halo; cervical spine decompression; corpectomy at C3; posterior fusion at C1-C4
Smucker et al. [12]	59	F	Neck pain; dysphagia; Raynaud’s phenomenon	Posterior calcification of C4-C5	posterior fusion at C3-C6
Smucker et al. [12]	73	F	Neck pain radiating to shoulders; BUE weakness; fine motor dysfunction	Listhesis at multiple levels in the cervical spine	Posterior fusion at C2-T4; anterior fusion at C3-T1
Ward et al. [13]	62	F	Neck pain radiating to occiput, ear; dysphagia; Raynaud’s phenomenon; limited range of motion	Calcific masses at C1-C3	N/A
Haverbush et al. [14]	46	F	Neck pain radiating to shoulder and arm; limited range of motion; dysphagia	Kyphosis and subluxation at C5-C6; calcifications at C3-C5	Posterior fusion at C1-C7
Present case	60	F	Neck pain; inability to ambulate; dysphagia	Cervical kyphosis; degeneration from C4-C6; disc bulging at C3-C4 and C5-C6; posterior osteophytes at C3-C4; uncovertebral osteophytes at C4-C5 and C5-C6	Posterior fusion at C2-T8; Ponte osteotomies at T2-T6

The patient may have developed cervical kyphosis secondary to diffuse scleroderma, but we must also consider the impact of age-related changes and degeneration. Previous case reports have described the presence of calcification as a contributing factor in the development of spinal instability and neurological sequelae in scleroderma patients. Calcinosis of the soft tissues can present in autoimmune diseases, such as scleroderma, systemic lupus erythematosus, and mixed connective tissue disorders, but can also occur along the cervical vertebrae [7]. Cervical calcinosis in scleroderma patients may derive from recurrent microtrauma of bony structures [17]. The cervical spine is prone to microtrauma secondary to its high flexibility, with certain areas more susceptible to degenerative disc disease and herniation [7,18-20]. Osteophytes, which can form in response to microtrauma, aging, and degeneration, were present from C3-C6 with posterior osteophytes at C3-C4 and uncovertebral osteophytes at C5-C6.

Although the spine maintains flexibility due to the presence of elastic ligaments and intervertebral discs, this flexibility may be impaired with disc degeneration and calcinosis. This patient had a four-month history of scleroderma and presented with pain, reduced range of motion, and cervical kyphosis. These symptoms may have resulted from the recent-onset active scleroderma with contributions from age-related changes or degeneration. This was supported by imaging studies, which showed disc degeneration, loss of disc height, disc bulging, and osteophyte formation along the kyphotic portion of the cervical spine. A non-contrast cervical CT scan also revealed spinal canal narrowing at C3-C5 and C6-C7 in addition to foraminal narrowing at C4-C6. Narrowing of these key portions of the vertebral column may lead to the entrapment of nerves, contributing to both motor and sensory deficits.

## Conclusions

While scleroderma is a complex autoimmune disorder that can present with a wide range of symptoms, cervical kyphosis is among the rarest of manifestations. Soft tissues are primarily affected in scleroderma patients, yet some may present with spinal abnormalities due to calcification along the vertebral column. We described the case of a 60-year-old female with a four-month history of scleroderma, who presented with cervical kyphosis, neck pain, difficulty with ambulation, dysphagia, edema, and reduced range of motion. The patient's recent-onset diffuse scleroderma, aging, and degeneration may have contributed to her neurological presentations and the development of cervical kyphosis. It is important to understand the varying symptoms in scleroderma patients to manage symptoms appropriately and mitigate further degeneration. This report provides insights into the unique presentation of cervical kyphosis in a scleroderma patient.
